# The Influence of Diet Composition on Fitness of the Blue Crab, *Callinectes sapidus*

**DOI:** 10.1371/journal.pone.0145481

**Published:** 2016-01-19

**Authors:** Benjamin A. Belgrad, Blaine D. Griffen

**Affiliations:** 1 School of Earth, Ocean, and Environment, Marine Science Program, University of South Carolina, Columbia, South Carolina, United States of America; 2 Department of Biological Sciences, University of South Carolina, Columbia, South Carolina, United States of America; Sonoma State University, UNITED STATES

## Abstract

The physiological condition and fecundity of an organism is frequently controlled by diet. As changes in environmental conditions often cause organisms to alter their foraging behavior, a comprehensive understanding of how diet influences the fitness of an individual is central to predicting the effect of environmental change on population dynamics. We experimentally manipulated the diet of the economically and ecologically important blue crab, *Callinectes sapidus*, to approximate the effects of a dietary shift from primarily animal to plant tissue, a phenomenon commonly documented in crabs. Crabs whose diet consisted exclusively of animal tissue had markedly lower mortality and consumed substantially more food than crabs whose diet consisted exclusively of seaweed. The quantity of food consumed had a significant positive influence on reproductive effort and long-term energy stores. Additionally, seaweed diets produced a three-fold decrease in hepatopancreas lipid content and a simultaneous two-fold increase in crab aggression when compared to an animal diet. Our results reveal that the consumption of animal tissue substantially enhanced *C*. *sapidus* fitness, and suggest that a dietary shift to plant tissue may reduce crab population growth by decreasing fecundity as well as increasing mortality. This study has implications for *C*. *sapidus* fisheries.

## Introduction

Numerous studies have found that individual diet and physiological well-being are interdependent. Diet plays a crucial role in the metabolic efficiency [1–2] and homeostasis of an organism [3]. Diet can also influence the accumulation and toxicity of heavy metals [4–5], and even affect the longevity [6–7] as well as the reproductive output of individuals [8–9]. Shifts in diet may therefore indirectly alter population dynamics by changing an individual’s longevity or offspring production. Indeed, dietary alterations have been proposed as the mechanism behind the population decline of some species of seabirds [10–11] and shore crabs [12], and for fluctuations in the abundance of trout [13].

Organisms may alter their diet for a variety of reasons. Ontogenetic dietary shifts are a widespread phenomenon [14–15]. Mature individuals frequently consume different prey than juveniles due to developmental changes which produce differences in size, competitive ability, and metabolic processes [14]. Diets may also shift in response to alterations in the environment. For instance, seasonal changes [16], climatic regime shifts [10, 17], and species invasions [18–19] can induce diet shifts either by introducing new species to consume or by altering the availability of native species. Diseases too can cause dietary shifts either by reducing the abundance of prey species [20] or by impairing the ability of the host to digest or capture prey [21]. Finally, pollution can cause dietary shifts by altering consumer behavior and/or reducing foraging capabilities. One such example has been found in the blue crab, *Callinectes sapidus* [22].

Normally the diet of blue crabs consists of 20–40% mollusks, 10–26% arthropods, 5–12% fishes, and 1–7% polychaetes [22–24]. Algae, sediment, and detritus can also compose a small percentage (~3%) of the diet under normal conditions [22–24]. However, crabs within estuaries contaminated with metals predominantly consume plant matter/algae (27%) and sediment/detritus (39%), and much less animal tissue overall (34%); presumably because of impaired coordination and reduced ability to capture active prey (e.g. fish; [22]). Metal pollution also reduces overall food consumption and causes crabs to exhibit more cannibalistic tendencies as well as abnormally aggressive behavior [22, 25]. Specific diets of individual crabs may also be influenced by numerous other factors, including food availability [26], individual preference [27], crab size [28], or physiological condition [24].

Blue crabs are a commercially important species which inhabit estuaries in the western Atlantic and can occur from Nova Scotia to northern Argentina [29–30]. The species has been harvested by commercial fisheries since the late 19^th^ century and today constitutes a multimillion dollar industry, becoming the largest crab fishery (by pound) in the United States [31–33]. Blue crabs are also an ecologically important species. They consume a wide variety of organisms across several phyla and also act as prey for more than 100 species [23–24]. Their predatory activities can have far-reaching consequences as fluctuations in bivalve mortality rates often coincide with blue crab abundance [34], and predation by blue crabs can control the structure of benthic infaunal communities [35]. Thus, the impacts of diet selection in this species on individual physiological performance and on fecundity can have important economic and ecological implications.

The purpose of this study was to investigate the relationship between diet, physiological condition, fecundity, and behavior in the blue crab, *C*. *sapidus*, in order to understand the importance of diet selection for blue crab population dynamics. We therefore experimentally manipulated the diet of crabs both qualitatively and quantitatively and measured resulting differences on crab mortality, reproductive potential (amount of tissue invested in reproduction and egg size), long-term energy stores (hepatopancreas size and lipid content), and aggression. Blue crabs were expected to have reduced reproductive potential and decreased energy stores from consuming seaweed diets since the crabs normally consume animal tissue primarily, while aggression was expected to increase with enhanced hunger levels.

## Methods

### Sampling and Holding

We collected 60 mature female *Callinectes sapidus* (mean ± SD carapace width = 14.5 ± 0.8 cm) that were not missing any limbs using baited crab traps from the North Inlet National Estuarine Research Reserve (33°20’N, 79°10’W, Georgetown, South Carolina). Field collections of blue crabs were conducted under a permit issued by the South Carolina Department of Natural Resources, and blue crabs are not an endangered species. Crabs were obtained during early May 2014 over the course of a week, one month prior to the peak spawning season [36–37]. We started the experiment in blocks (5 total) as crabs were captured so that no crabs were held longer than 24 hours before commencing the study and being fed.

Crabs were transported to the Baruch Institute wet lab (situated on North Inlet) where they were placed into individual plastic containers (length 29.8 cm, width 19.7 cm, height 20.3 cm) submersed within seven flow-through tanks supplied with seawater directly from North Inlet. Water temperature matched environmental conditions and varied between 25.4–34.5°C throughout the experiment. Individual containers were filled with a 1.5 cm layer of sediment collected from the field and continuously received water at a rate of ~1.3 L/min. Once a week the containers were cleaned with an aquarium vacuum and the substrate layer was replenished with new sediment. This sediment was provided because sediment is required for development of normal egg masses in this species [38]. Sediment may also have served as an additional source of food, though sediment consumption was not measured. Crabs that died before the end of the experiment were frozen and stored at -20°C for later dissection. Any egg masses produced by the crabs were stored in the freezer for later analyses. The experiment was terminated after 12 weeks, on 30-Jul-2014 when surviving crabs were frozen for later dissection. No molting occurred during the course of this study and no crabs died or produced broods until over two weeks after the experiment began.

### Feeding

Throughout the duration of the experiment, crabs were fed either exclusively ribbed mussels (*Geukensia demissa*; 2.09 kJ/g wet weight [39]), mummichogs (*Fundulus heteroclitus*; 4.23 kJ/g wet weight [40]), or seaweed (*Ulva lactuca*; 1.60 kJ/g wet weight [41]), with all crabs having access to sediment, to isolate the effects of each food type and determine the maximum change in fitness induced by a dietary shift. We collected *G*. *demissa*, *F*. *heteroclitus*, and *U*. *lactuca* from our field site daily to ensure crabs were provided natural fresh food sources. Each of these species is common throughout salt marshes and all are frequently consumed by blue crabs [22–24]. Because consumers are known to compensate for low-quality diets by increasing the amount of food consumed [42–43], we fed crabs either a satiating amount of food (4 ribbed mussels, 25.2 g mummichog, 3.7 g seaweed) or approximately one-quarter this amount (1 ribbed mussel, 5.8 g mummichog, 1.3 g seaweed). The quantities of food offered depended on food type. While mummichog weight corresponded to the average weight of the soft tissue within 4 or 1 mussels, seaweed weight related to the volume of 25.2 or 5.8 g of mummichog because *U*. *lactuca* is substantially less dense than mummichog and the amount of food blue crabs can consume is dependent on their stomach capacity [44]. Thus, this study had a 3x2 factorial design (i.e. food type x portion size) with ten crabs randomly assigned to each of the six different experimental diets. Two weeks after the original 60 crabs were caught; four additional mature female crabs were collected and starved for two months in chambers with sediment to compare the effects of starvation to our food treatments. Starved crabs were excluded from all statistical analyses due to the lower number of replicates and later collection date, but were included in figures as visual references.

Crabs were fed a constant experimental diet every other day and any excess food was removed after 24 h. Mussels were cracked open prior to being fed to the crabs in an effort to make handling effort more similar across food types, and only soft tissue weights of mussels were used in analyses. A generalized linear model (GLM) with a binomial distribution was employed to determine if either the food type (mussel, fish, or seaweed) or amount of food offered (large or small portions) influenced crab mortality. All statistical analyses were conducted in R, version 3.0.2 (R Development Core Team, Auckland, New Zealand). We originally included experimental block and holding tank as blocking factors in the statistical models described below, however, these were not significant and so data were pooled across blocks and tanks for all analyses.

### Behavior Measurements

We assessed individual crab aggression levels daily to determine if diet influenced behavior. To reduce biases in behavior originating from previous feeding history we did not begin measuring behavior until two weeks after capture. Behavior was measured by slowly lowering a metal prong (25.0 cm x 0.5 cm) into each container, stopping approximately three cm from the mouth of the crab, and observing the crab response. The container sides were opaque to help prevent the crab from reacting to stimuli outside of the container and the observer was careful to never appear directly over the container. Similar techniques have been used previously to examine the aggressive behavior of animals (e.g. squid [45]). Crab behavior was categorized as aggressive if the crab approached or raised its chelipeds towards the prong, while stationary crabs or crabs that moved away from the prong were labeled as docile. Crab behavior was measured once each day between the hours 1200–1330 prior to feeding to help control for any behaviors associated with the crab circadian rhythm and to prevent changes in behavior associated with consuming food. We examined the factors that influenced crab behavior using a mixed—effects GLM with a binomial distribution. The response variable in this analysis was crab behavior on each sampling day (aggressive or docile). We treated food type, portion size offered, time since last fed (24 or 48 h), and daily temperature as fixed factors, and individual crab ID as a random factor to control for repeated measures of each individual crab. For presentation purposes only, data are shown as the proportion of observations where crabs were aggressive for each factor.

### Tissue Analyses

At the end of the experiment, crabs were dissected and the primary energy storage organ of crabs, the hepatopancreas [46], was removed to assess the relative physiological condition of individuals. Similarly, both the ovaries and developing eggs were removed. These were combined with any egg masses the crab produced during the experiment to determine the amount of tissue crabs invested in reproduction. The hepatopancreas, reproductive tissues, and remainder of the crab were dried separately to constant weight at 70°C. The mass of the hepatopancres was divided by the dry mass of the rest of the crab to produce a size independent index of long-term energy stores (hepatosomatic index; HSI) following the protocol of [47]. Likewise, the mass of the reproductive tissue underwent an analogous calculation to produce a size independent index of reproductive effort (gonadosomatic index; GSI). We performed separate 2-way ANOVAs to determine how food type and portion size offered influenced HSI and GSI, followed by Tukey’s multiple comparison tests. Prior to this and all subsequent statistical analyses, Shapiro—Wilk tests of normality and homogeneity of variance were conducted.

We also assessed long term energy storage in terms of hepatopancreas lipids. We determined the bulk lipid content of the hepatopancreas using a modified Folch method where chloroform was replaced with hexanes [48–49]. We determined the percent lipid composition of the hepatopancreas by dividing the dry weight of the extracted lipids with the initial hepatopancreas dry weight. In order to clearly present the relationship between hepatopancreas condition and diet, we conducted a 2-way ANOVA to assess the impact of food type and portion size offered on the percent lipid of the hepatopancreas as well as a linear model II regression correlating the HSI of the hepatopancreas to the % lipids of the hepatopancreas.

Crab oocytes were analyzed by rehydrating subsamples of eggs from each crab using filtered seawater and photographed under a dissecting microscope to determine the average egg volume (μm^3^). Ten eggs were randomly selected from each crab, and the areas of the eggs were computed using the software SIGMA Photo Pro version 5.5.2. This allowed us to back-calculate the volume of the eggs by applying the equation for a sphere. To roughly estimate the number of eggs produced by each crab, we calculated the average mass of an individual egg using the previously determined egg volumes for each crab and assuming that eggs had the same density as water. The total number of eggs generated was obtained by dividing the overall mass of the eggs with the estimated mass of a single egg. Given the unverified assumptions within these calculations, we only use these estimates to compare the relative number of eggs produced between individuals (since identical assumptions were applied across all individuals). Separate 2-way ANOVAs were used to determine how food type and portion size offered influenced the size and calculated amount of eggs produced. Tukey’s multiple comparison tests were used to determine pairwise differences.

## Results

### Mortality

In total, six crabs fed seaweed died while only one crab fed mussels died and there was no mortality in crabs fed a fish diet. Crabs fed seaweed were found to have significantly higher mortality than crabs fed either fish or mussels (GLM, df = 3, t = -2.908, *p* = 0.0052), but the amount of food offered did not significantly alter mortality (GLM, df = 3, t = 0.268, *p* = 0.7893).

### Behavior

**Fig 1:**
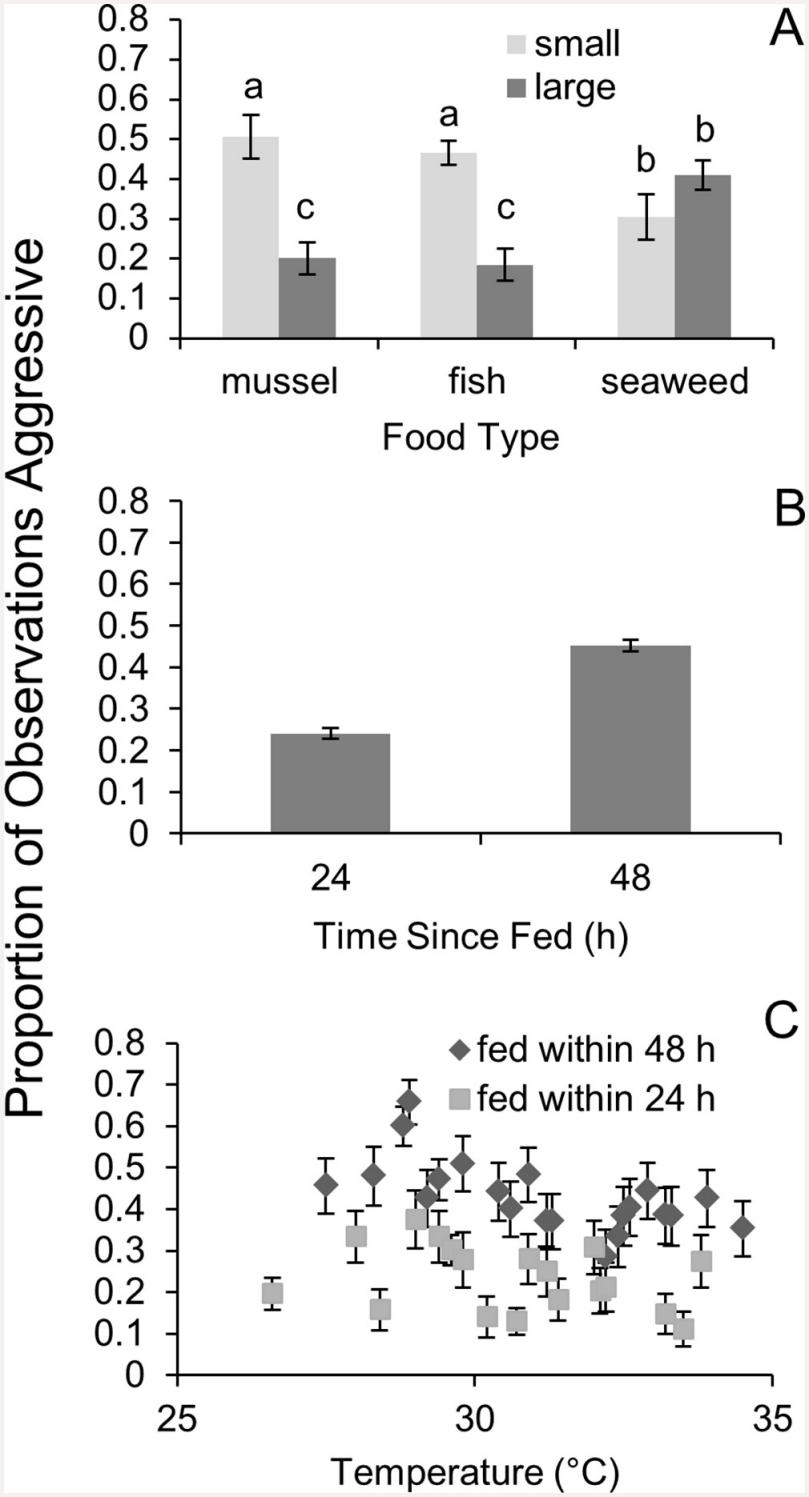
Analysis of crab behavior. Mean ± SE aggression probability of crabs as a function of **A)** diet (n = 10), **B)** time since last feeding (24 or 48 h; n = 1168 and 1163 respectively), and **C)** daily temperature (°C) within holding containers for crabs fed within 24 or 48 h (n = 53–159). Replicates depend on the number of surviving crabs and times exposed to the same temperature (max = 3 same temperature).

Both food type and amount of food offered significantly affected crab behavior. Crabs which consumed seaweed were over twice as likely to be aggressive (41% of time) as compared to crabs which consumed animal matter (aggressive19% of time) (mixed—effects GLM; Z = 3.86, *p* = 0.0011), while there was no significant difference in behavior between crabs which consumed either mussels or fish (mixed—effects GLM; Z = 0.51, *p* = 0.6096; Fig 1A). Crab aggression levels decreased as food portion size increased (mixed—effects GLM; Z = 4.95, *p* < 0.0001). We found a significant interaction between food type and portion size on behavior (mixed effects GLM; Z = -4.18, *p* < 0.0001) so that crabs which consumed seaweed exhibited higher aggression when fed more (Fig 1A).

Additionally, crab aggression significantly varied with temperature (mixed—effects GLM; Z = -4.98, *p* < 0.0001; Fig 1B). Time since feeding also influenced aggression, with crabs aggressive 24% of the time 24 h after feeding and 45% of the time 48 h after feeding (mixed—effects GLM; Z = 2.07 *p* = 0.0383; Fig 1C). Aggressive behavior was also consistent through time for individuals, regardless of experimental conditions as indicated by the significant random effect of individual (Chi-square = 107.25 comparing model with and without random effect, df = 1, *p* < 0.0001).

### Tissue Analyses: Energy Storage

**Fig 2:**
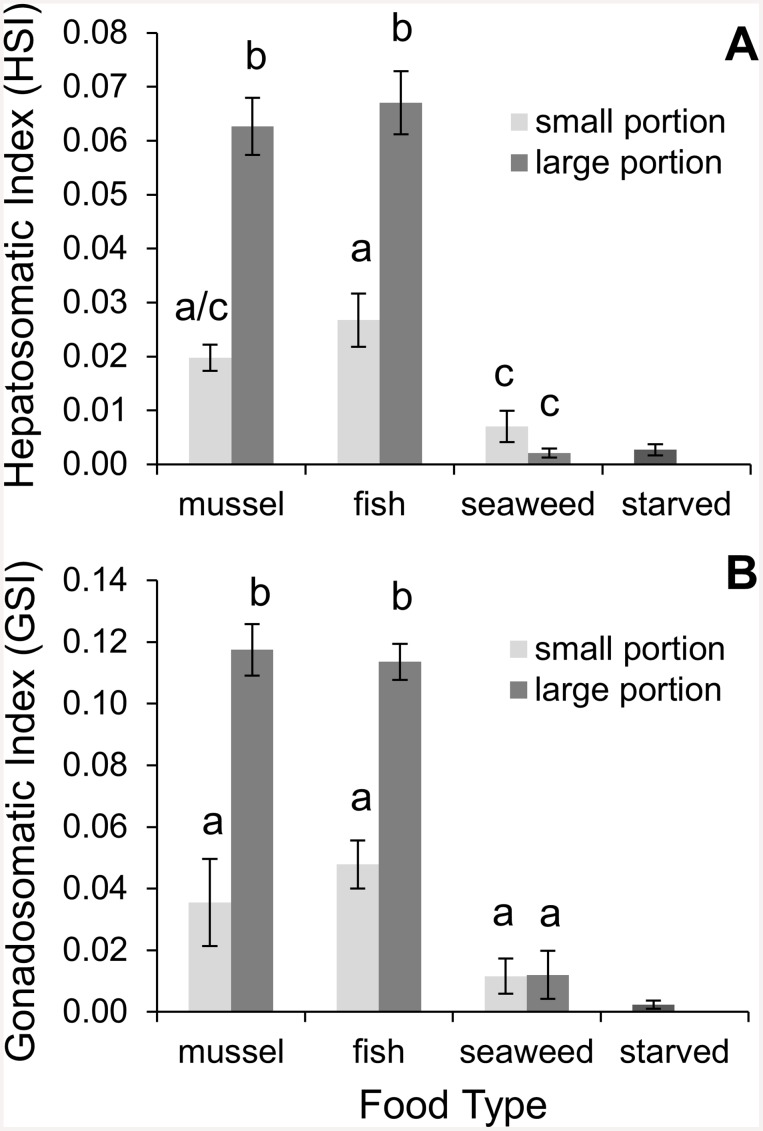
Effect of diet on tissue investment towards the hepatopancreas and reproduction. Mean ± SE **A)** Hepatosomatic Index (HSI) and **B)** gonadosomatic Index (GSI) of crabs fed either ribbed mussels (*Geukensia demissa*), fish (*Fundulus heteroclitus*), or seaweed (*Ulva lactuca*) at one of two portion sizes (large or small) for ~2.5 months (n = 10). For reference purposes, starved crabs were expressed in the figures as separate columns (n = 4). Starved crabs were not used in the statistical analyses.

Diet had a strong influence on crab energy stores. The physiological condition of crabs as denoted by HSI was significantly affected by food type and portion size (2-way ANOVA; food type: F = 60.19, df = 2, *p* < 0.0001; portion size: F = 58.31, df = 1, *p* < 0.0001). Crabs fed large portions of animal matter stored on average three times more energy than crabs fed small portions of animal matter, and over 14 times more than crabs fed seaweed (Fig 2A). Food type and portion size interacted so crabs fed seaweed produced the same size energy stores regardless of portion size (2-way ANOVA; F = 20.60, df = 2, *p* < 0.0001; Table 1).

**Table 1:** Analysis of Tukey’s HSD multiple comparison test to determine pairwise differences in the gonadosomatic index (GSI), hepatosomatic index (HSI), egg volume, and egg number of crabs given different diets.

Comparison	GSI*p*-value	HIS*p*-value	Egg Volume*p*-value	Egg Number*p*-value
Mussel Large—Fish Large	0.9996	0.9764	0.4247	0.8976
Seaweed Large—Fish Large	*< 0*.*0001*	*< 0*.*0001*	*0*.*0001*	*0*.*0023*
Fish Small—Fish Large	*< 0*.*0001*	*< 0*.*0001*	0.1034	0.4299
Mussel Small—Fish Large	*< 0*.*0001*	*< 0*.*0001*	*0*.*0109*	0.3108
Seaweed Small—Fish Large	*< 0*.*0001*	*< 0*.*0001*	*0*.*0003*	*0*.*0014*
Seaweed Large—Mussel Large	*< 0*.*0001*	*< 0*.*0001*	*0*.*0302*	*<0*.*0001*
Fish Small—Mussel Large	*< 0*.*0001*	*< 0*.*0001*	0.9675	*0*.*0452*
Mussel Small—Mussel Large	*< 0*.*0001*	*< 0*.*0001*	0.4770	*0*.*0308*
Seaweed Small—Mussel Large	*< 0*.*0001*	*< 0*.*0001*	*0*.*0467*	*<0*.*0001*
Fish Small—Seaweed Large	0.0578	*0*.*0015*	0.1873	0.2152
Mussel Small—Seaweed Large	0.4145	*0*.*0472*	0.8428	0.4407
Seaweed Small—Seaweed Large	1.000	0.9615	0.9999	0.9999
Mussel Small—Fish Small	0.9167	0.8448	0.8985	0.9995
Seaweed Small—Fish Small	0.0533	*0*.*0185*	0.2840	0.1612
Seaweed Small—Mussel Small	0.3951	0.2772	0.9160	0.3589

Diet altered food type (ribbed mussels, *Geukensia demissa*; mummichogs, *Fundulus heteroclitus*; seaweed, *Ulva lactuca*) and portion size (large, small). Significant values are in italics.

**Fig 3:**
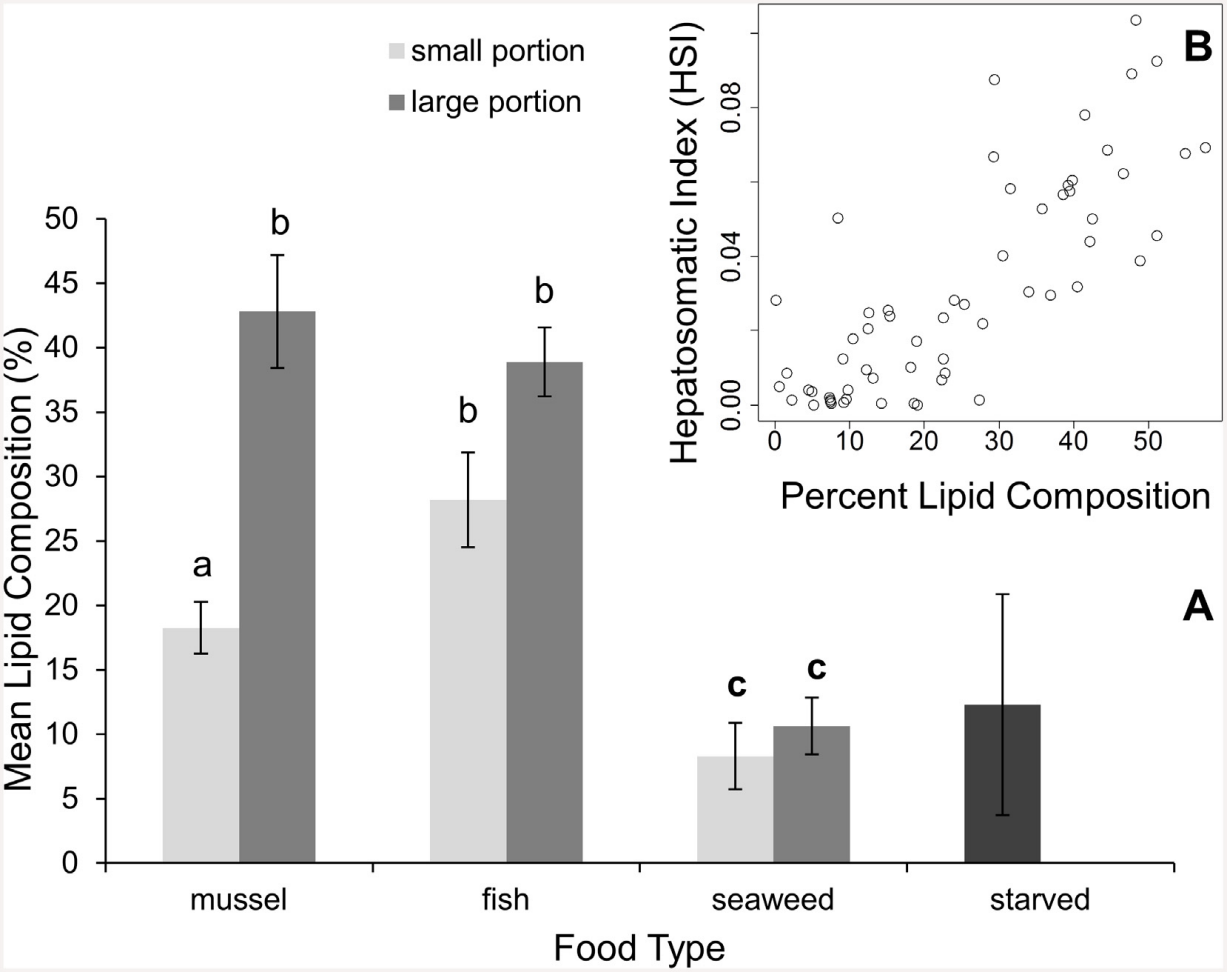
A) Analysis of percent lipid composition of the hepatopancreas. The effect of food type (ribbed mussels, *Geukensia demissa*; mummichogs, *Fundulus heteroclitus*; seaweed, *Ulva lactuca*) and portion size offered (large, small) on the mean ± SE percent lipid composition of the crab hepatopancreas (n = 10). Starved crabs (n = 4) were represented in the figure to serve as a visual reference and were not included in the statistical analysis. Lower case letters denote statistical differences (*p* < 0.001, 2-way ANOVA, Tukey test). **B)** The relationship of percent lipid composition of the hepatopancreas for individual crabs and their corresponding hepatosomatic index (HSI, n = 60).

**Fig 4:**
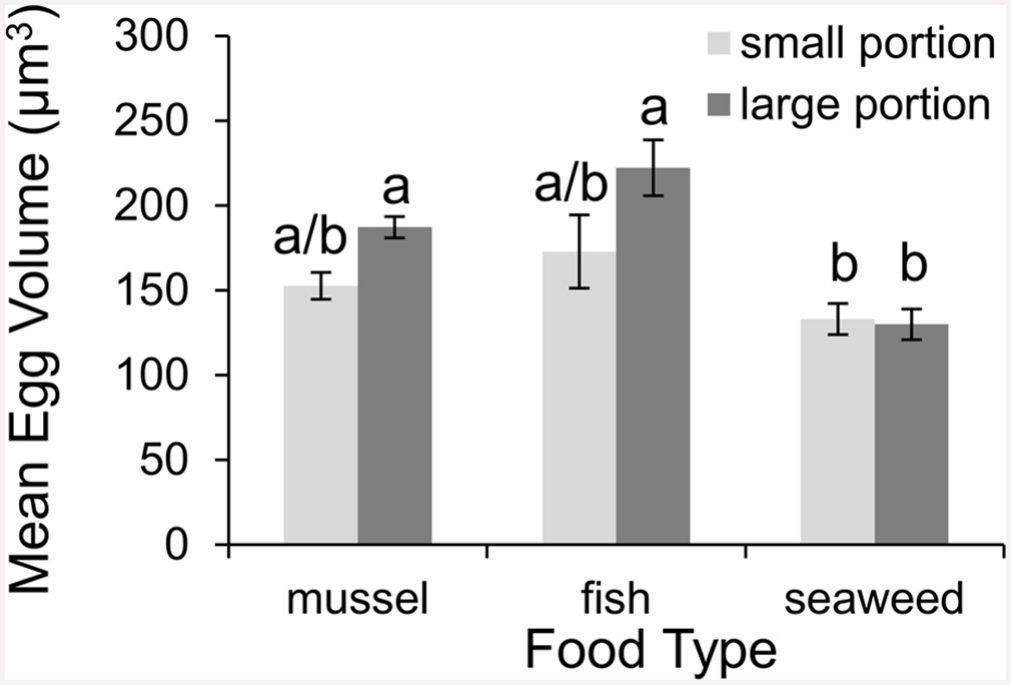
Effect of diet on egg size. Mean ± SE egg volume (μm^3^) of crabs fed either ribbed mussels (*Geukensia demissa*), fish (*Fundulus heteroclitus*), or seaweed (*Ulva lactuca*) at one of two portion sizes (large or small) for ~2.5 months (n = 10).

Similar patterns were observed in terms of lipid storage, as both food type and amount of food offered interacted to influence the lipid content of the hepatopancreas (2-way ANOVA; df = 2, F = 6.12, *p* = 0.0039; Fig 3A). The hepatopancreas lipid content for crabs fed animal matter was over three times higher than for crabs fed seaweed, regardless of portion size (2-way ANOVA; df = 3, F = 24.09, *p* < 0.0001). On average, the lipid content of the hepatopancreas of crabs fed large portions of food was 67% higher than for crabs fed small portions of food (Fig 4A). However, only crabs fed mussels had a significant difference in lipid content between portion sizes (2-way ANOVA; df = 1, F = 22.90, *p* < 0.0001) The lipid content of the hepatopancreas and HSI were strongly correlated as lipid content explained 64% of the variation in HSI (linear regression; df = 58, t = 10.16, *p* < 0.0001; Fig 3B). In other words, crab hepatopancreases became larger with an increase in their lipid composition.

### Tissue Analyses: Reproductive Potential

Similar to the physiological condition of crabs, the reproductive effort of crabs as indicated by GSI was also significantly affected by both food type and portion size (2-way ANOVA; food type: F = 39.02 df = 2, *p* < 0.0001; portion size: F = 47.89 df = 1, *p* < 0.0001). Crabs fed large portions of animal matter invested on average nearly three times more towards reproduction than crabs fed small portions of animal matter, and almost 10 times the amount invested by crabs fed seaweed (Fig 2B). Food type and portion size interacted so crabs that consumed seaweed invested the same amount of tissue towards reproduction regardless of portion size (2-way ANOVA; F = 12.21, df = 2, *p* < 0.0001; see Table 1 for pairwise comparisons).

The mean volume of eggs crabs produced also depended upon both food type and portion size (2-way ANOVA; food type: F = 12.41, df = 2, *p* < 0.0001; portion size: F = 6.09, df = 1, *p* = 0.0171). Although there was not a significant difference in egg size between crabs fed the same portions of fish and mussels, crabs fed large portions of animal matter produced eggs 25% larger than crabs given small portions of animal matter, and 55% larger than crabs given seaweed (Fig 4; Table 1). Food type and portion size were not found to interact (2-way ANOVA; F = 2.105, df = 2, *p* < 0.1325). Likewise, both food type and portion size regulated the relative number of eggs crabs produced (2-way ANOVA; food type: F = 16.41, df = 2, *p* < 0.0002; portion size: F = 8.91, df = 1, *p* = 0.0044). Crabs produced almost three times more eggs when given large portions of animal matter than when given seaweed or small portions of animal matter. Food type and portion size did not interact to influence egg numbers (2-way ANOVA; F = 2.29, df = 2, *p* = 0.1116).

## Discussion

By experimentally controlling the diet of the commercially harvested blue crab, *Callinectes sapidus*, we demonstrate that diet has a strong impact on crab mortality, fecundity, physiological condition, and behavior. *C*. *sapidus* fed seaweed invested significantly less tissue in reproduction and internal energy stores, and exhibited substantially higher mortality and aggression, than crabs fed animal matter. Studies on the carnivorous rock crab *Cancer irroratus* [50], on the omnivorous European green crab *Carcinus maenas* [51], and on the herbivorous mangrove tree crab *Aratus pisonii* [47] all report similar findings across the dietary continuum from carnivores to herbivores—that increased consumption of animal tissue improves the fecundity and physiological condition of crabs. These results have important implications for individuals and populations that switch from consuming primarily animal tissue to diets that consist predominantly of algae and plant matter.

Seaweed diets may have reduced crab fitness through several non-mutually exclusive processes. Although blue crabs frequently consume *Ulva lactuca*, seaweed normally constitutes less than 10% of the material blue crabs consume in healthy ecosystems [24, 52]. Exclusive consumption of *U*. *lactuca* in this experiment could have reduced fitness via the buildup of toxic exudates [53] or by providing insufficient nutrition. Seaweed has high levels of indigestible material (~20% cellulose and hemicellulose) as well as relatively low levels of nitrogen and lipids compared to animal tissue [54–56]. Nitrogen limitation in particular is a common phenomenon among herbivorous crabs [54].

### Behavior

Crab aggression levels increased with the consumption of seaweed and decreased with portion size. Seaweed may have increased crab aggression either through hormonal changes or by partially starving the crabs. Leopoldo *et al*. [57] report that the amino acid tryptophan can suppress the aggressiveness of mud crabs, whereas Hazlett *et al*. [58] and Stocker and Huber [59] have documented increased aggression in crustaceans under starvation conditions. These two mechanisms may have worked in concert to raise crab aggression since *U*. *lactuca* has relatively low concentrations of tryptophan compared to animal tissue and other protein sources [60]. More research will be necessary to separate the relative influence of starvation and tryptophan on crab behavior; although the increase in crab aggression during each 48 h feeding period coupled with physiological condition implies the primary driving force for the behavior change is starvation.

### Tissue Analyses

The hepatopancreas is the main energy storage organ of crustaceans, serving as the primary storage site of lipids (long-term energy stores) as well as one of several storage sites of glycogen (short-term energy stores; [46]). Depleted hepatopancreas lipid stores are indicative of starvation [61], and have been suggested to reduce reproduction [62]. The consumption of seaweed probably reduced crab energy stores because typically the lipid content of seaweed is less than 4% [55] and *U*. *lactuca* in particular has a lipid content below 0.5% [63]. Similarly, crabs fed small portions of mussels likely exhibited significantly lower lipid stores than crabs fed fish because ribbed mussels contain considerably less lipids (~5%) as a proportion of dry weight than mummichogs (~10%) [64–65]. Although crabs can build lipid stores through lipid anabolism from excess proteins and carbohydrates, the process is not as efficient as the direct uptake of lipids through lipid rich diets [66]. These differences in the lipid content of the food and corresponding decline in lipid storage of the hepatopancreas are likely responsible for the observed decrease in reproductive effort because the size and amount of eggs produced depends on the availability of lipids [67]. A portion of the GSI as measured here was comprised of extruded eggs, while the remainder was comprised of vitellogenic ovaries. Smaller vitellogenic ovaries should translate directly into lower reproductive output in *C*. *sapidus*, as the amount of eggs crabs produce is directly proportional to gonad mass [51, 68]. Furthermore, larval mortality in crustaceans and fish is negatively correlated to egg size [69–71]. This implies that crabs which consume seaweed should yield larvae with higher mortality than crabs which consume animal matter because crabs fed seaweed generally produced eggs 30% smaller than crabs fed animal matter. Crabs may have compensated for increasing the size of their eggs by decreasing the amount of eggs generated. However, a rough calculation of the number of eggs each crab produced determined that brood size increased with the amount and type of food consumed. In fact, calculated egg size and egg number were positively correlated, meaning that crabs simultaneously increased both egg quality and number when their diet improved, consistent with patterns in other crab species [47]. Such a pattern was likely observed because the improved diet provided more energy and nutrients for crabs to invest towards reproduction.

### Fishery Implications

These findings have implications for blue crab fisheries, as the experimental diet shifts imposed here (algal consumption and lower consumption overall) are similar to diets documented by Reichmuth et al. [22] for crabs in estuaries that are heavily contaminated by heavy metals. The increased mortality coupled with the decreased reproductive potential and energy reserves of crabs consuming seaweed substantially reduced their fitness in comparison to crabs consuming animal tissue. Thus, the dietary shift documented by Reichmuth et al. [22] potentially causes the *C*. *sapidus* population of metal polluted estuaries to experience lower population growth than populations within clean estuaries. Indeed, our study presents a conservative estimate of the impacts of metal pollution because we only examined the implications of diet shift alone, and these are probably further exacerbated when the toxic effects of the pollutant that caused the diet shift are taken into account. It should be recognized that our results depict the maximum change in fitness induced by a dietary shift, since we examined the effects of pure animal and pure plant diets. By contrast, crabs experiencing metal pollution shift their diets towards greater herbivory, but still ingest some animal matter [22]. Our results suggest that decreasing the amount of animal matter consumed, without entirely eliminating it, can still have a substantial impact on fitness. Diet mixing can be an effective strategy and is known to increase fitness relative to single diets [72]. However, previous work across a range of crab species indicates that mixing plant and animal foods in the diet does not offer any benefit for fecundity or energy storage relative to carnivorous diets alone [47, 50–51, 73]. Diet mixing or shifting from animal tissue to predominantly seaweed diets by crabs within polluted estuaries may alternatively benefit crabs by helping reduce the amount of metals crabs accumulate, since toxins can biomagnify up trophic levels [74]. However, most marine invertebrates primarily accumulate toxins by uptake from the surrounding water column [75]. Many other types of pollutants besides metals, ranging from pesticides to polycyclic aromatic hydrocarbons (PAHs; fossil fuel derivatives), are known to impair the foraging behavior of crabs and fish [76–78], and may indirectly reduce the fitness of exposed species through such dietary changes.

### Broader implications for ecology

The research presented here has at least two broader implications for population ecology. First, this study underscores the importance of examining the nonlethal effects of environmental stressors that cause diet shifts. For instance, our results demonstrate that the reproductive effort and physiological condition of crabs may decrease substantially from the indirect effects of pollutants, independent of any direct effects from the pollutants. While many studies document physiological and behavioral changes in response to contaminants [79–80], relatively few studies have explored how these indirect effects may alter the fitness of organisms (but see [81–83]. The tight link between diet and fitness also reveals the necessity of including dietary shifts when predicting population responses to environmental change. Many current environmental issues such as climate change [10, 84] and species invasions [18] are accompanied with drastic changes in diet. However, when calculating the community and population response to these changes, the indirect effect of dietary shifts are frequently either ignored or considered too complex to incorporate [85]. Our results imply that, when indirect effects from diet shifts are included, these environmental changes may have larger effects than previously anticipated.

Second, the positive correlation between the number of eggs produced and their size shows that individuals may improve offspring quantity and quality simultaneously. The well-known r- and K-selection theory postulates that species try to maximize fitness by producing either a large amount of low quality offspring or a small amount of high quality offspring depending on their life history strategy [85]. As energy stores are finite, species must trade-off between quantity and quality [86–87]. In contrast, our findings suggest that during times of abundant resources some organisms will bet-hedge by simultaneously employing both options: increasing egg quantity and enhancing egg quality.

In conclusion, fecundity, physiological condition, and behavior are significantly influenced by diet. Our experiments show that the fitness of an important fishery species was enhanced with increased consumption of animal tissue while the consumption of seaweed reduced the fecundity and long-term energy stores as well as increased the mortality and aggression of *C*. *sapidus*. This study reveals the impacts that diet selection can have on individuals’ performance and on the potential for population growth of this important fishery species.

## References

[1] Carew LB, Hopkins DT, Nesheim MC. Influence of amount and type of fat on metabolic efficiency of energy utilization by the chick. J Nutr. 1964; 83: 300–306. 14194428 10.1093/jn/83.4.300

[2] Karowe DN, Martin MM. The effects of quantity and quality of diet nitrogen on the growth, efficiency of food utilization, nitrogen budget, and metabolic rate of fifth-instar *Spodoptera eridania* larvae (Lepidoptera: noctuidae). J Insect Physiol. 1989; 35: 699–708.

[3] Zhang L, Bruce-Keller AJ, Dasuri K, Nguyen A, Liu Y, Keller JN. Diet-induced metabolic disturbances as modulators of brain homeostasis. Biochim Biophys Acta. 2009; 1792: 417–422. 10.1016/j.bbadis.2008.09.006 18926905 PMC2735018

[4] Peraza MA, Ayala-Fierro F, Barber DS, Casarez E, Rael LT. Effects of micronutrients on metal toxicity. Environ Health Persp. 1998; 106: 203–216. 10.1289/ehp.98106s1203 PMC1533267 9539014

[5] Saric MM, Blanusa M, Piasek M, Varnai VM, Juresa D, Kostial K. Effects of dietary calcium on cadmium absorption and retention in suckling rats. Biometals. 2002; 15: 175–182. 12046926 10.1023/a:1015212929481

[6] Bishop NA, Guarente L. Two neurons mediate diet-restriction-induced longevity in *C*. *elegans*. Nature. 2007; 447: 545–549. 17538612 10.1038/nature05904

[7] Mair W, Dillin A. Aging and survival: The genetics of life span extension by dietary restriction. Annu Rev Biochem. 2008; 77: 727–754. 10.1146/annurev.biochem.77.061206.171059 18373439

[8] Xu X L, Ji WJ, Castell JD, O’Dor RK. Influence of dietary lipid sources on fecundity, egg hatchability, and fatty composition of Chinese prawns (*Penaeus chinensis*) broodstock. Aquaculture. 1994; 119: 359–370.

[9] Jorgensen HB, Toft S. Food preference, diet dependent fecundity and larval development in *Harpalus rufipes* (Coleoptera: Carabidae). Pedobiologia. 1997; 41: 307–515.

[10] Kitaysky AS, Kitaiskaia EV, Piatt JF, Wingfield JC. A mechanistic link between chick diet and decline in seabirds?. P. Roy Soc B. 2006; 273: 445–450. 10.1098/rspb.2005.3351 PMC1560207 16615211

[11] Norris DY, Arcese P, Preikshot D, Bertram DF, Kyser TK. Diet reconstruction and historic population dynamics in a threatened seabird. J Appl Ecol. 2007; 44: 875–884.

[12] Griffen BD, Altman I, Hurley J, Mosblack H. Reduced fecundity by one invader in the presence of another: a potential mechanism leading to species replacement. J Exp Mar Biol Ecol. 2011; 406: 6–13.

[13] Kelly DW, Dick J A. 2005 Introduction of the nonindigenous amphipod *Gammarus pulex* alters population dynamics and diet of juvenile trout *Salmo trutta*. Freshwater Biology, 50: 127–140.

[14] Lucifora LO, Garcia VB., Menni RC, Escalante AH, Hozbar NM. Effects of body size, age, and maturity stage on diet in a large shark: Ecological and applied implications. Ecol Res. 2009; 24: 109–118.

[15] Davis AM, Pearson RG, Pusey BJ, Perna C, Morgan DL, Burrows D. Trophic ecology of northern Australia’s terapontids: Ontogenetic dietary shifts and feeding classification. J Fish Biol. 2011; 78: 265–286. 10.1111/j.1095-8649.2010.02862.x 21235560

[16] Rosalino LM, Loureiro F, Macdonald DW, Santon-Reis M. Dietary shifts of the badger (*Meles meles*) in Mediterranean woodlands: An opportunistic forager with seasonal specialisms. Z Saugetierkd. 2005; 70: 12–23.

[17] Decker MB, Hunt GL, Byrd GV. The relationships between sea-surface temperature, the abundance of juvenile walleye pollock (*Theragra chalcogramma*) and the reproductive performance and diets of seabirds at the Pribilof Islands, southeastern Bering Sea In: Beamish RJ, editor. Climate Change and Northern Fish Populations. Ontario: National Research Council of Canada; 1995 pp. 425–437.

[18] Eagles-Smith CA, Suchanek TH, Colwell AE, Anderson NL, Moyle PB. Changes in fish diets and food web mercury bioaccumulation induced by an invasive planktivorous fish. Ecol Appl. 2008; 18: A213–A226. 19475926 10.1890/06-1415.1

[19] Griffen BD, Guy T, Buck JC. Inhibition between invasives: a newly introduced predator moderates the impacts of a previously established invasive predator. J Anim Ecol. 2008; 77: 32–40. 10.1111/j.1365-2656.2007.01304.x 18177327

[20] Moleon M, Sanchez-Zapata JA, Real J, Garcia-Charton JA, Gil-Sanchez JM, Palma L, et al Large-scale spatio-temporal shifts in the diet of a predator mediated by an emerging infectious disease of its main prey. J Biogeogr. 2009; 36: 1502–1515.

[21] Shields JD. Diseases of spiny lobsters: A review. J Invertebr Pathol. 2011; 106: 79–91. 10.1016/j.jip.2010.09.015 21215357

[22] Reichmuth JM, Roudez R, Glover T, Weis JS. Differences in prey capture behavior in populations of blue crab (*Callinectes sapidus* Rathbun) from contaminated and clean estuaries in New Jersey. Estuaries Coasts. 2009; 32: 298–308.

[23] Laughlin RA. Feeding habits of the blue crab, *Callinectes sapidus* Rathbun, in the Apalachicola Estuary, Florida. B Mar Sci. 1982; 32: 807–822.

[24] Hines AH. Ecology of juvenile and adult blue crabs In: Kennedy VS, Cronin LE, editors. The Blue Crab, *Callinectes sapidus*. College Park: Maryland Sea Grant College; 2007 pp. 565–654.

[25] Reichmuth JM, MacDonald J, Ramirez J, Weis JS. Fight or flight: An investigation of aggressive behavior and predator avoidance in two populations of blue crabs (*Callinectes sapidus* Rathbun) in New Jersey. Hydrobiologia. 2011; 658: 173–182.

[26] Blundon JA, Kennedy VS. Mechanical and behavioral aspects of the blue crab, *Callinectes sapidus* (Rathbun), predation on Chesapeake Bay bivalves. J Exp Mar Biol Ecol. 1982; 65: 47–65.

[27] Micheli F. Behavioral plasticity in prey-size selectivity of the blue crab *Callinectes sapidus* feeding on bivalve prey. J Anim Ecol. 1995; 64: 63–74.

[28] Arnold WS. The effects of prey size, predator size, and sediment composition on the rate of predation of the blue crab, *Callinectes sapidus* Rathbun, on the hard clam, *Mercenaria mercenaria* (Linne). J Exp Mar Biol Ecol. 1984; 80: 207–219.

[29] Williams AB. Swimming crabs of the genus *Callinectes* (Decapoda: Portunidae). Fish B. 1974; 72: 685–798.

[30] Millikin MR, Williams AB. Synopsis of biological data on the blue crab, *Callinectes sapidus* Rathbun. In: NOAA Technical Report NMFS 1, FAO Fisheries Synopsis No. 138. Silver Spring: 1984. pp. 1–32.

[31] Rathbun R. The common edible or blue crab: *Callinectes hastatus*, Ordway In: Goode GB, editor. The Fisheries and Fishery Industries of the United States, Section I, Part V, Y—Crustaceans, Article 222. Washington DC: US Commission of Fish and Fisheries; 1884 pp. 775–778.

[32] Rathbun R. The crab, lobster, crayfish, rock lobster, shrimp, and prawn fisheries, 1(a) 1. Natural history and uses of the blue crab In: Goode GB, editor. The Fisheries and Fishery Industries of the United States, Section V, Volume II, Part XXI. Washington DC: US Commission of Fish and Fisheries; 1887 pp. 629–648.

[33] NMFS (National Marine Fisheries Service) Fisheries of the United States 2012. Silver Spring: National Marine Fisheries Service Fisheries Statistics Division; 2013. Available: http://www.st.nmfs.noaa.gov/Assets/commercial/fus/fus12/02_commercial2012.pdf. Accessed 19 February 2014.

[34] Fiorenza M. Effects of predator foraging behavior on patterns of prey mortality in soft marine bottoms. Ecol Monogr. 1999; 67: 203–224.

[35] Virnstein RW. The importance of predation by crabs and fishes on benthic infauna in Chesapeake Bay. Ecology. 1977; 58: 1199–1217.

[36] Dickinson GH, Rittschof D, Latanich C. Spawning biology of the blue crab, *Callinectes sapidus*, in North Carolina. B Mar Sci. 2006; 79: 273–285.

[37] Darnell MZ, Rittschof D, Darnell KM, McDowell RE. 2009. Lifetime reproductive potential of female blue crabs *Callinectes sapidus* in North Carolina, USA. Mar Ecol Prog Ser. 2009; 394: 153–163.

[38] Zmora O, Findiesen A, Stubblefield J, Frenkel V, Zohar Y. Large-scale juvenile production of the blue crab *Callinectes sapidus*. Aquaculture. 2005; 244: 129–139.

[39] McKinney RA, Glatt SM, McWilliams SR. Allometric length-weight relationships for benthic prey of aquatic wildlife in coastal marine habitats. Wildl Biol. 2004; 10: 241–249.

[40] Dunn EH. Caloric intake of nestling double-crested cormorants. Auk. 1975; 92: 553–565.

[41] Shpigel M, Ragg NL, Lupatsch I, Neori A. Protein content determines the nutritional value of the seaweed *Ulva lactuca* L for the abalone *Haliotis tuberculata* L. and *H*. *discus hannai* Ino. J Shellfish Res. 1999; 18: 227–233.

[42] Simpson SJ, Simpson CL. The mechanism of nutritional compensation by phytophagous insects In: Bernays EA, editor. Insect—Plant Interactions. Vol 2 Boca Raton: CRC Press; 1990 pp. 111–160.

[43] Simpson SJ, Raubenheimer D, Chambers PG. The mechanisms of nutritional homeostasis In: Regulatory Mechanisms in Insect Feeding, Chapman RF, de Boer G, editors. New York: Springer; 1995 pp. 251–278.

[44] Griffen BD, Mosblack H. Predicting diet and consumption rate differences between and within species using gut ecomorphology. J Anim Ecol. 2011; 80: 854–863. 10.1111/j.1365-2656.2011.01832.x 21418211

[45] Sinn DL, Moltschaniwskyj NA, Wapstra E, Dall SRX. Are behavioral syndromes invariant? Spatiotemporal variation in shy/bold behavior in squid. Behav Ecol Sociobiol. 2010; 64: 693–702.

[46] Parvathy K. Glycogen storage in relation to the moltcycle in two crustaceans *Emerita asiatica* and *Ligia exotica*. Mar Biol. 1971; 10: 82–86.

[47] Riley ME, Vogel M, Griffen BD. Fitness-associated consequences of an omnivorous diet for the mangrove tree crab, *Aratus pisonii*. Aquat Biol. 2014; 20: 35–43.

[48] Pickova J, Dutta PC, Larsson PO, Kiessling A. Early embryonic cleavage pattern, hatching success and egg-lipid fatty acid composition. Can J Fish Aquat Sci. 1997; 54: 2410–2416.

[49] Undeland I, Harrod M, Lingnert H. Comparison between methods using low-toxicity solvents for the extraction of lipids from herring. Food Chem 1998; 61: 355–365.

[50] Griffen BD, Riley ME. Potential impacts of invasive crabs on one life history strategy of native rock crabs in the Gulf of Maine. Biol Inv. 2015; 17: 2533–2544.

[51] Griffen BD. Linking individual diet variation and fecundity in an omnivorous marine consumer. Oecologia. 2014; 174: 121–130. 10.1007/s00442-013-2751-3 23996228

[52] Dittel AI, Epifanio CE, Fogel ML. Trophic relationships of juvenile blue crabs (*Callinectes sapidus*) in estuarine habitats. Hydrobiologia. 2006; 568: 379–390.

[53] Johnson DA, Welsh BL. Detrimental effects of *Ulva lactuca* (L.) exudates and low oxygen on estuarine crab larvae. J Exp Mar Biol Ecol. 1985; 86: 73–83.

[54] Wolcott D, O’Connor N. Herbivory in crabs: Adaptations and ecological considerations. Am Zool. 1992; 32: 370–381.

[55] Herbetreau F, Coiffard LJM, Derrien A, De RoeckHolzhauer Y. The fatty acid composition of five species of macroalgae. Bot Mar. 1997; 40: 25–27,

[56] Linton SM, Greenaway P. A review of feeding and nutrition of herbivorous land crabs: Adaptations to low quality plant diets. J Comp Physiol B. 2007; 177: 269–286. 17279390 10.1007/s00360-006-0138-z

[57] Leopoldo J, Laranja Q Jr, Quinitio ET, Catacutan MR, Coloso RM. Effects of dietary L-tryptophan on the agonistic behavior, growth, and survival of juvenile mud crab *Scylla serrata*. Aquaculture. 2010; 310: 84–90.

[58] Hazlett B, Rubenstein D, Rittschof D. Starvation, energy reserves, and aggression in the crayfish *Orconectes virilis* (Hagen, 1870) (Decapoda, Cambaridae). Crustaceana. 1975; 28: 11–16.

[59] Stocker AM, Huber R. Fighting strategies in crayfish *Orconectes rusticus* (Decapoda, Cambaridae) differ with hunger state and the presence of food cues. Ethology. 2001; 107: 727–736.

[60] Vinoj Kumar V, Kaladharan P. Amino acids in the seaweeds as an alternate source of protein for animal feed. J Mar Biol Assoc India. 2007; 49: 35–40.

[61] Sanchez-Paz A, Garcia-Carreno F, Hernandez-Lopez J, Muhlia-Almazan A, Yepiz-Plascenia G. Effect of short-term starvation on hepatopancreas and plasma energy reserves of the Pacific white shrimp (*Litopenaeus vannamei*). J Exp Mar Biol Ecol. 2007; 340: 184–193.

[62] Vazquez Boucard CG, Patrois J, Ceccaldi HJ. Exhaustion of lipid reserves in the hepatopancreas of *Fenneropenaeus indicus* broodstock in relation to successive spawnings. Aquaculture. 2004; 236: 523–537.

[63] Ortiz J, Romero N, Robert P, Araya J, Lopez-Herandez J, Bozzo C. et al Dietary fiber, amino acid, fatty acid, and tocopherol contents of the edible seaweeds *Ulva lactuca* and *Durvillaea antartica*. Food Chem. 2006; 99: 98–104.

[64] Bergen BJ, Nelson WG, Quinn JG, Jayaraman S. Relationships among total lipid, lipid classes, and polychlorinated biphenyl concentrations in two indigenous populations of ribbed mussels (*Geukensia demissa*) over an annual cycle. Environ Toxicol Chem. 2001; 20: 575–581. 11349859

[65] Weinstein MP, Litvin SY, Guida VG. Essential fish habitat and wetland restoration success: A tier III approach to the biochemical condition of common mummichog *Fundulus heteroclitus* in common reed *Phragmites australis*- and smooth cordgrass *Spartina alterniflora*- dominated salt marshes. Estuaries Coasts. 2009; 32: 1011–1022.

[66] Kucharski LCR, Da Silva RSM. Effect of diet composition on the carbohydrate and lipid metabolism in an estuarine crab, *Chasmagnathus granulate* (Dana, 1851). Comp Biochem Physiol A. 1991; 99: 215–218.

[67] Doughty P, Shine R. Detecting life history trade-offs: measuring energy stores in “capital” breeders reveals costs of reproduction. Oecologia. 1997; 110: 508–513. 28307242 10.1007/s004420050187

[68] Hartnoll RG. Reproductive investment in Brachyura. Hydrobiologia. 2006; 557: 31–40.

[69] Bagenal TB. Relationship between egg size and fry survival in brown trout *Salmo trutta* L. J Fish Biol. 1969; 1: 349–353.

[70] Gimenez L, Anger K. Relationships among salinity, egg size, embryonic development, and larval biomass in the estuarine crab *Chasmagnathus granulate* Dana, 1851. J Exp Mar Biol Ecol. 2001; 260: 241–257. 11358581 10.1016/s0022-0981(01)00258-1

[71] Gimenez L, Anger K. Larval performance in an estuarine crab, *Chasmagnathus granulate*, is a consequence of both larval and embryonic experience. Mar Ecol Prog Ser. 2003 249: 251–264.

[72] Coll M, Guershon M. Omnivory in terrestrial arthropods: Mixing plant and prey diets. Annu Rev Entomol. 2002; 47: 267–297. 11729076 10.1146/annurev.ento.47.091201.145209

[73] Griffen BD, Norelli AP. Spatially variable habitat quality contributes to within-population variation in reproductive success. Ecology Evol. 2015; 5: 1474–1483. 10.1002/ece3.1427 PMC4395176 25897386

[74] Guthrie RK, Davis EM, Cherry DS, Murray HE. Biomagnification of heavy metals by organisms in a marine microcosm. B Environ Contam Tox. 1979; 21: 53–61. 10.1007/BF01685385 444708

[75] Gray JS. Biomagnification in marine systems: The perspective of an ecologist. Mar Pollut Bull. 2002; 45: 46–52. 12398366 10.1016/s0025-326x(01)00323-x

[76] Little EE, Archeski RD, Flerov BA, Kozlovskaya VI. Behavioral indicators of sublethal toxicity in rainbow trout. Arch Environ Con Tox. 1990; 19: 380–385. 10.1007/BF01054982 2353837

[77] Weis JS, Smith G, Zhou T, Santiago-Bass C, Weis P. Effects of contaminants on behavior: Biochemical mechanisms and ecological consequences. Bioscience. 2001; 51: 209–217.

[78] Dissanayake A, Piggott C, Baldwin C, Sloman KA. Elucidating cellular and behavioral effects of contaminant impact (polycyclic aromatic hydrocarbons, PAHs) in both laboratory-exposed and field-collected shore crabs Carcinus maenas (Crustacea: Decapoda). Mar Environ Res. 2010; 70: 368–373. 10.1016/j.marenvres.2010.07.004 20727579

[79] Fleeger JW, Carman KR, Nisbet RM. Indirect effects of contaminants in aquatic ecosystems. Sci Total Environ. 2003; 317: 207–233. 14630423 10.1016/S0048-9697(03)00141-4

[80] Macneale KH, Kiffney PM, Scholz NL. Pesticides, aquatic food webs, and the conservation of Pacific salmon. Front Ecol Environ. 2010; 8: 475–482.

[81] Relyea RA, Diecks N. An unforeseen chain of effects: Lethal effects of pesticides on frogs at sublethal concentrations. Ecol Appl. 2008; 18: 1728–1742. 18839767 10.1890/08-0454.1

[82] Duquesne S, Leiss M. Indirect effects of pesticides on mosquito larvae via alterations of community structure. Israel J Ecol Evol. 2010; 56: 433–447.

[83] Hanlon SM, Relyea R. Sublethal effects of pesticides on predator-prey interactions in amphibians. Copeia. 2013; 4: 691–698.

[84] Montevecchi WA, Myers RA. Centurial and decadal oceanographic influences on changes in northern gannet populations and diets in the north-west Atlantic: Implications for climate change. ICES J Mar Sci. 1997; 54: 608–614.

[85] McBride MM, Dalpadado P, Drinkwater KF, Godo OR, Hobday AJ, Hollowed AB, et al Krill, climate, and contrasting future scenarios for Arctic and Antarctic fisheries. ICES J Mar Sci. 2014; 71: 1934–1955.

[86] Pianka ER. On r- and K-selection. Am Nat. 1970; 104: 592–597.

[87] Smith CC, Fretwell SD. The optimal balance between size and number of offspring. Am Nat. 1974; 108: 499–506.

